# Eating Behavior in Children Aged 3–6: Relationship With the Child's Temperament Characteristics and Parent's Feeding Style

**DOI:** 10.1002/brb3.70758

**Published:** 2025-08-12

**Authors:** İbrahim Zeyrek, Esra Güney, Ahmet Özaslan, Asiye Uğraş Dikmen

**Affiliations:** ^1^ Department of Child and Adolescent Psychiatry Memorial Diyarbakır Hospital Diyarbakır Turkey; ^2^ Department of Child and Adolescent Psychiatry Gazi University Medical Faculty Ankara Turkey; ^3^ Psychology Research Centre Khazar University Baku Azerbaijan; ^4^ Department of Public Health Gazi University Medical Faculty Ankara Turkey

**Keywords:** eating behavior, feeding style, temperament

## Abstract

**Objective:**

To explore the relationship between children's eating behaviors, parental feeding behaviors, and child temperament among Turkish preschoolers aged 3–6 years.

**Methods:**

A cross‐sectional survey study was conducted between September 2021 and January 2022. Participants included 203 parents of preschool‐aged children (aged 3–6 years) attending nursery and kindergartens, as well as those seeking help from the Department of Child and Adolescent Psychiatry. Analyses were performed utilizing IBM SPSS Statistics version 23.0. Categorical variables are summarized in tables, showing counts and percentages in the descriptive statistics section. The association between continuous variables was assessed through Spearman correlation analysis. In this study, a significance level of *p* < 0.05 was established.

**Results:**

Emotional feeding was positively associated with emotional overeating and a desire to drink in children (*r* = 0.316, *p* < 0.01; *r* = 0.266, *p* < 0.01). Emotional overeating correlated negatively with instrumental feeding (*r* = −0.158, *p* < 0.05) but positively with encouragement feeding (*r* = 0.215, *p* < 0.01). The temperamental trait of surgency was positively associated with the desire to drink (*r* = 0.177, *p* < 0.05) and satiety responsiveness (*r* = 0.226, *p* < 0.01). Negative affect correlated negatively with enjoyment of food (*r* = −0.255, *p* < 0.01) and food fussiness (*r* = −0.225, *p* < 0.01) but positively with satiety responsiveness (*r* = 0.347, *p* < 0.01) and slowness in eating (*r* = 0.282, *p* < 0.01). Effortful control exhibited negative associations with emotional overeating (*r* = −0.207, *p* < 0.01) and the desire to drink (*r* = −0.141, *p* < 0.05).

**Conclusions:**

This study identifies significant associations between specific parental feeding behaviors and children's eating habits, along with the influence of child temperament traits on these behaviors. Key findings highlight the positive relationship between emotional feeding and children's emotional overeating, along with the significant negative impacts of effortful control on emotional overeating and the desire to drink. Other noteworthy results include the strong negative association between negative affect and enjoyment of food, as well as its positive correlation with satiety responsiveness and slowness in eating. These results suggest that both parental feeding strategies and individual temperamental traits significantly influence preschoolers’ eating behaviors. Interventions tailored to enhance parental feeding styles while considering children's temperaments may promote healthier eating practices in young children.

## Graphical abstract

Higher instrumental feeding tightly contolled feeding and surgency were associated with greater food responsiveness. Higher emotional feeding increased emotional overeating. This study identifies significant associations between specific parental behaviors and children's eating habits, along with the influence of temperament traits on these behaviors.

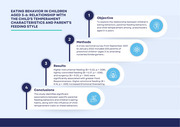

## 1 Introduction

Eating habit‐related issues are prevalent among children aged 3–6 years. Previous studies have reported a general prevalence of eating problems in this age group ranging from 26.9% to 37.2% (de SousaMaranhão et al. 2017; Benjasuwantep et al. 2013). Research has indicated that the period between ages 3 and 6 is of paramount significance for establishing lifelong healthy eating habits and represents a critical window for preventing childhood obesity (Juanjuan 2017; Shloim et al. 2015). Given the high prevalence of childhood obesity, it is crucial to prioritize the investigation of the factors impacting children's eating behaviors (Lobstein et al. 2004). In children, both temperament and parental feeding style play significant roles in children's eating behavior (Haycraft et al. 2011; Yuan et al. 2019). These factors can influence eating behaviors and contribute to eating problems and obesity in children. It is widely recognized that establishing healthy eating habits during the preschool period significantly influences eating behaviors during school years and adulthood (Ünlü 2011). Since healthy eating behavior begins to develop in early childhood, studying the parental feeding style and temperament can provide insights into a child's eating behavior (Morin 2006).

### 1.1 Parental Feeding Style

Parents serve as crucial influencers in the development of children's food preferences and eating styles, ranging from controlling a child's food intake to passively modeling a healthy or unhealthy diet (Larsen et al. 2015). Understanding the connections between caregivers' feeding practices and children's eating behaviors is valuable for creating tailored interventions and enhancing parental guidance for addressing unhealthy eating habits in preschool‐aged children (Yuan et al. 2019). The way parents impact their child's feeding behavior is multifaceted and can occur through their own feeding style. The three extensively studied aspects of parental feeding style include coercion, restraint, and instrumental feeding (IF). Pressure is often applied to encourage the consumption of healthier foods, or sometimes simply to consume more, particularly during mealtimes. Restriction is frequently aimed at unhealthy foods, especially energy‐dense snacks (Birch et al. 2001). IF involves using food as a reward. In contrast, emotional feeding (EM), which involves offering food to manage children's negative mood states, has garnered attention due to retrospective reports suggesting its practice by parents of obese patients (Wardle et al. 2002). Several studies have found associations: maternal restriction linked to child food responsiveness (FR), and maternal pressure to eat associated with child satiety responsiveness (SR), slowness, fussiness, and anorexia (Gregory et al. 2010; Nowicka et al. 2014; Webber et al. 2010). Furthermore, it has been demonstrated that caregivers' feeding behaviors can be associated with childhood overweight and obesity by influencing children's eating behaviors (Yuan et al. 2019).

### 1.2 Temperament

Temperament refers to a collection of inherent traits that manifest as relatively consistent behavioral tendencies in reaction to specific situations. In simpler terms, it can be seen as the qualities that form an individual's behavioral patterns or their inherent tendencies to act in particular ways. Temperamental aspects that have been suggested as potential factors affecting eating behaviors include impulsivity (Braet et al. 2007), surgency (Vollrath et al. 2011), negative emotionality (Haycraft et al. 2011; Vollrath et al. 2011; Anzman‐Frasca et al. 2012), and self‐regulation, which also involves effortful control (Anzman‐Frasca et al. 2012). Rothbart characterizes these three overarching dimensions of temperament as surgency, negative affectivity, and effortful control. These dimensions are seen as representing individual variations in reactivity and self‐regulation, which are biologically rooted and relatively consistent across different situations (Rothbart et al. 2000). Surgency represents a temperament dimension characterized not just by impulsiveness but also by a strong desire for intense pleasure, a high level of activity, and a low propensity for shyness (Rothbart and Putnam 2002). The temperament dimension known as negative affectivity is defined by mood instability, a tendency toward angry reactions, and difficulty regulating negative emotions (Shields and Cicchetti 1997). Effortful control as a temperament dimension is marked by the ability to restrain oneself from engaging in a preferred or dominant behavior, all the while sustaining focus on a task and resisting distractions (Rothbart and Putnam 2002).

These innate inclinations may offer insight into why some individuals develop obesity in their current environment while others do not (Anzman‐Frasca et al. 2012). Temperament traits have been associated with obesity and excess weight in children, as well as with problematic eating attitudes and behaviors in individuals ranging from infants to adolescents and adults. To illustrate, emotional temperament in children has been linked to the emergence of excess weight, and it has been observed as a mediator in the connection between a child's weight status and that of their parents (Agras et al. 2004). Difficult infant temperament has been linked to negative mealtimes, food refusal in young children, and the use of negative feeding practices by parents (Farrow and Blissett 2006). Higher levels of emotional temperaments have been linked to less enjoyment of food (EF), greater fussy eating, slower eating, higher SR, and increased emotional overeating (EOE) and undereating (Haycraft et al. 2011). Parent reports also indicated that children with shy temperament traits were more likely to exhibit reluctance in trying new foods (Pliner and Loewen 1997). Furthermore, a study examining child temperament traits and overweight or obesity found a positive association between negative affectivity in infants and preschoolers and overweight or obesity (Bergmeier et al. 2015).

Previous research has established links between temperament and eating behaviors in preschool children. Children's food approach behaviors encompass FR, EOE, the EF, and a desire to drink (DD). On the other hand, food avoidance behaviors include SR, slow eating, emotional undereating (EUE), and food fussiness (FF). Emotional eating (EE) refers to a change in eating behavior in response to distress, resulting in either increased food intake (overeating) or avoidance of food (undereating). EE has been found to be associated with temperament, specifically negative emotionality (Messerli‐Bürgy et al. 2018). Difficult temperament traits, such as difficulty in calming down, negative mood, and low adaptability, have been linked to negative mealtimes, food rejection, and negative feeding practices by parents in young children (Farrow and Blissett 2006; Blissett and Farrow 2007). Furthermore, feeding difficulties were found to be more prevalent in children with difficult temperaments characterized by higher levels of negative affect and reactivity, or in demanding children. Higher reactivity and low regulation have shown associations with parental/caregiver feeding practices conducive to obesity, children's eating behaviors, and weight‐related outcomes in infancy and early childhood (Anzman‐Frasca et al. 2012; Bergmeier et al. 2014; McMeekin et al. 2013; Leung et al. 2014).

Upon reviewing the available literature, it becomes evident that the eating behaviors of children during the preschool growth and development period can be influenced directly or indirectly by numerous factors, including the child's temperament and parental feeding style. Understanding the connections between children's eating behaviors and the factors that impact their eating behavior is essential for developing effective interventions to address unhealthy eating behaviors. This knowledge also contributes to parents' ability to manage unhealthy eating behaviors in children aged 3–6 years. This study states that although there are some studies showing that temperament and parental feeding style are performed with older adolescent or adult samples as well as studies on eating behavior in children, the literature examining these relationships in preschool children and investigating all dimensions of temperament is insufficient. Considering that it is a period when preschool children's autonomy over eating becomes somewhat more pronounced, this study aims to fill this gap in the literature by first examining the relationships between parent‐reported young children's temperaments and parental feeding style, and typical eating behaviors assessed using well‐established measurement tools. In addition, this study aims to contribute to the literature by examining the relationship between all temperament characteristics and parental feeding styles and eating behavior in children.

This study comprehensively examined the complex relationships between children's eating behaviors, parental feeding practices, and child temperament, particularly in the context of Turkish preschool children aged 3–6 years. Although previous research has examined these variables, most of the existing literature is based on Western populations, leaving a gap in understanding how cultural factors influence these dynamics in Turkey. In addition to contributing to the growing body of knowledge on child nutrition and development, this study also highlights the importance of cultural context in shaping eating behaviors. Furthermore, the inclusion of child temperament in the analysis adds a new dimension that allows for a more nuanced understanding of how individual differences between children may influence their interactions with food and parental guidance. This research has potential implications for tailored interventions that consider both cultural and individual factors and thus improve feeding practices among young children in Turkey and potentially outside Turkey.

Regarding the initial aim of the study, the hypothesis was that children with a more difficult temperament (for example, more discomfort or less soothability) were thought to have more food avoidance and less food‐approaching behavior (for example, higher levels of SR and lower levels of food enjoyment). For the second aim, the hypothesis was that children with a higher EM would exhibit more food‐approaching behaviors (for example, higher FR and EOE) and a higher control feeding would exhibit less food‐approaching behaviors (e.g., less levels of overeating and food enjoyment).

## 2 Materials and Methods

### 2.1 Subjects

The study was conducted between September 2021 and January 2022 and involved parents with children aged 3–6 who sought assistance from the Department of Child and Adolescent Psychiatry, as well as children aged 3–6 attending nursery and kindergartens. The study was conducted at Gazi University, Department of Child and Adolescent Psychiatry, Ankara, TURKEY. This study was approved by the Gazi University Ethics Committee on 03.08.2021 with approval number 2021–811.

The inclusion criteria for the subjects were as follows: (1) Having a child between the ages of 3–6 in preschool. (2) Obtaining parental consent after receiving information about the study. The exclusion criteria were as follows: (1) The child had previously sought psychiatric help or received psychological counseling for any eating problems. (2) The child had any systemic diseases (e.g., asthma). (3) The parent did not provide consent to participate in the study. A total of 222 parents with children aged 3–6 participated in the study. Following the application of the inclusion/exclusion criteria, 19 parents were excluded, and the study proceeded with 203 parents. The participation of parents in the study was voluntary.

### 2.2 Measures

#### 2.2.1 Sociodemographic Data Form

In the questionnaire designed for this study, demographic characteristics such as age, gender, education, and marital status of the parents were recorded. Descriptive details, including “use of feeding bottles,” “number of main meals,” “number of snacks,” and “transition to solid foods,” were also collected to aid in the assessment. Child body mass index (BMI) *Z* score values were calculated based on weight and height data provided by parents, using the criteria determined by Neyzi. The form was used to identify any systemic diseases mentioned in the exclusion criteria and to ascertain whether the child had a history of psychiatric admission.

#### 2.2.2 Parent Feeding Style Questionnaire (PFSQ)

The PFSQ is a 27‐item parent‐report measure that assesses parent feeding practices (Wardle et al. 2002). The PFSQ includes four subscales: EM (e.g., “I give my child something to eat to make him/her feel better when he/she has been hurt”), IF (e.g., “I reward my child with something to eat when he/she is well behaved”), prompting/encouragement (e.g., “I praise my child if he/she eats a new food”), and control over eating (e.g., “I decide how many snacks my child should have”). Response options include “I never do; I rarely do; I sometimes do; I often do; and I always do,” and scores range from 1 to 5 with higher scores reflecting a higher frequency of the feeding behavior occurring. The PFSQ has demonstrated adequate internal consistency and test–retest reliability (Wardle et al. 2002). In the current study, PFSQ subscales demonstrated good internal consistency: EM *α* = 0.88, IF *α* = 0.78, control *α* = 0.76, and encouragement *α* = 0.76. Its Turkish adaptation was carried out by Özçetin et al. In the Turkish version, controlled overeating is divided into two parts as tightly controlled overeating and tolerance controlled overeating (Özçetin et al. 2010). In this study, we have utilized the PFSQ to assess parent feeding practices of children.

#### 2.2.3 Children's Eating Behavior Questionnaire (CEBQ)

CEBQ is a 35‐item questionnaire that examines children's food approach and food avoidance eating behaviors developed by Wardle et al. (Wardle et al. 2001). The food approach subscales are as follows: FR, EOE, EF, and DD. The four food avoidant subscales are as follows: SR, slowness in eating (SE), EUE, and FF. Questions are responded to on a 5‐point Likert scale (never to always) and five items are reverse scored. Mean scores are calculated from the responses to each subscale and possible scores range from 1 to 5. Higher scores indicate a greater prevalence of that particular eating behavior. The CEBQ has been found to display good internal validity and reliability when completed by parents of young children (Wardle et al. 2001). In the current sample, Cronbach's alpha values are all good, ranging from 0.70 to 0.89. It was adapted into Turkish by Yılmaz et al. (Yılmaz et al. 2011). In this study, we have utilized the CEBQ to assess food approach and food avoidance eating behaviors of children.

#### 2.2.4 Child Behavior Questionnaire (CBQ)

CBQ is a widely used and validated 7‐point Likert‐type parent‐report measure assessing facets of temperament in children (where 1 is *extremely untrue* and 7 is *extremely true*) (Putnam and Rothbart 2006). We used the very short form version, which yields three factoranalytically derived subscales, Negative Affect, Surgency, and Effortful Control based on the original longer form CBQ. The very short form is validated in children 3–8 years of age (Putnam and Rothbart 2006). Higher scores of Surgency reflect greater global impulsivity and hedonic drive. Higher scores of Effortful Control reflect greater self‐control. Finally, higher scores of Negative Affect reflect greater global experience of negative emotions and poorer ability to self‐soothe. Rothbart and colleagues reported Cronbach alpha coefficients of 0.78, 0.69, and 0.74, respectively, for these constructs (Sleddens et al. 2012). The Turkish reliability and validity study of the CBQ was conducted by Sarı et al. (Sarı et al. 2012). In this study, we have utilized the CBQ to assess temperaments of children.

### 2.3 Procedure

The data for this study were collected using a questionnaire created through Google Forms, which included all the planned scales and data forms. The sociodemographic data form, CBQ, CEBQ, and PFSQ, were all incorporated into the Google form. The survey link was provided directly to parents who sought counseling at the Gazi University Faculty of Medicine, Department of Child and Adolescent Psychiatry, and met the inclusion criteria. For parents of children attending kindergartens affiliated with the Directorate of National Education, communication was established through online messaging platforms (e.g., WhatsApp) and email, facilitated by kindergarten teachers. Participants who met any of the exclusion criteria identified in the sociodemographic characteristics form were excluded from the study. Prior to participation, all participants were electronically provided with informed consent. The informed consent page presented two options (yes/no), and those who chose “yes” were directed to the page where the survey questions began.

### 2.4 Statistical Analysis

Considering that the results obtained from small sample sizes cannot be generalized to other samples, Green's (1991) criteria for determining the number of participants were adapted. Green recommends using at least 200 participants for any regression analysis, regardless of the number of predictor variables in the regression model. Therefore, it was planned to reach the highest level of participants to represent the universe in the sample with at least 200 participants (Green 1991).

Analyses were conducted using IBM SPSS Statistics version 23.0. In the descriptive statistics section, categorical variables are presented in tabular form by giving numbers and percentages. The relationship between continuous variables was evaluated using Spearman correlation analysis. The assumptions of multiple regression analyses—including linearity, homoscedasticity, multivariate normality, and absence of multicollinearity—were tested and confirmed to be satisfactory. No significant violations were observed. Multiple linear regression analyses were conducted to explore the predictive roles of parenting feeding practices and child temperament traits on various domains of children's eating behavior. Each eating behavior subscale was entered as a dependent variable in separate models. Independent variables included parental feeding strategies (e.g., EM, IF, encouragement, and tightly controlled feeding [TIC]/tolerance‐controlled feeding [TOC]), temperament dimensions (surgency, negative affect, and effortful control), and demographic variables (age and sex). The proportion of variance explained by each model was assessed using *R* ^2^ values. In this study, the statistical significance level was accepted as *p* < 0.05.

## 3 Results

### 3.1 Child Demographic Characteristics of the Subject

The mean age of 203 children evaluated in the study was 4.61 ± 0.92 years. Note that 47.8% (*n* = 97) of the children were male and 52.2% (*n* = 106) were female. Other results are shown in Table 1.

**TABLE 1:** Child demographic characteristics of the subject.

		Number	(%)
Child sex (*n* = 203)			
	Female	106	52.2
	Male	97	47.8
Child age (*n* = 203)			
	3	26	12.8
	4	63	31.1
	5	77	37.9
	6	37	18.2
Birth order (*n* = 203)			
	1	121	59.6
	2	47	23.2
	3 and above	35	17.2
Number of siblings (*n* = 203)			
	1	114	56.1
	2	59	29.1
	3 and above	30	14.8
Child weight status (*n* = 203)			
	Underweight	33	16.3
	Normal weight	110	54.1
	Overweight	15	7.4
	Obesity	45	22.2
Kindergarten status (*n* = 203)			
	Yes	154	75.9
	No	49	24.1

### 3.2 Parents Demographic Characteristics of the Subject

The mean age of the mothers of the children participating in the study was 33.1 ± 4.6 years, and the mean age of the fathers was 36.2 ± 5.0 years. Other parents demographic characteristics results are shown in Table 2.

**TABLE 2:** Parents demographic characteristics of the subject.

		Number	(%)
Maternal education (*n* = 203)			
	Primary school	12	5.9
	Middle school	14	6.9
	High school	48	23.7
	University	129	63.5
Maternal working status (*n* = 203)			
	Not working	132	65.0
	Working	71	35.0
Maternal illness status (*n* = 203)			
	Yes	16	7.9
	No	187	92.1
Maternal drug use status (*n* = 203)			
	Yes	31	15.3
	No	172	84.7
Paternal education (*n* = 203)			
	Primary school	8	3.9
	Middle school	15	7.4
	High school	43	21.2
	University	137	67.5
Paternal working status (*n* = 203)			
	Not working	5	2.5
	Working	198	97.5
Paternal illness status (*n* = 203)			
	Yes	19	9.4
	No	184	90.6
Paternal drug use status (*n* = 203)			
	Yes	24	11.8
	No	179	88.2
Family structure (*n* = 203)			
	Parents together	190	93.6
	Parents divorced	8	3.9
	Extended family	4	2.0
	Others	1	0.5
Monthly income (*n* = 203)			
	Low	8	3.9
	Medium	107	52.7
	High	88	43.4

### 3.3 Correlation Between Children's Eating Behavior and Temperament and Parent Feeding Behaviors

In the study, correlations between eight variables from CEBQ, five variables from PFSQ, and three variables from CBQ were analyzed and multiple regression analysis was performed to understand these relationships in more detail and their interactions with each other. The results of the Spearman correlation test are shown in supporting information tables.

**FIGURE 1:**
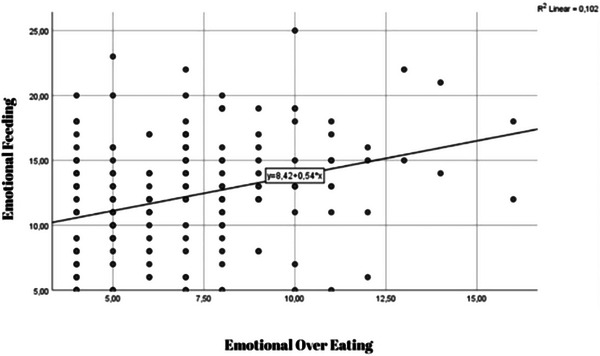
Scatter plot showing the positive association between Emotional Overeating and Emotional Feeding.

**FIGURE 2:**
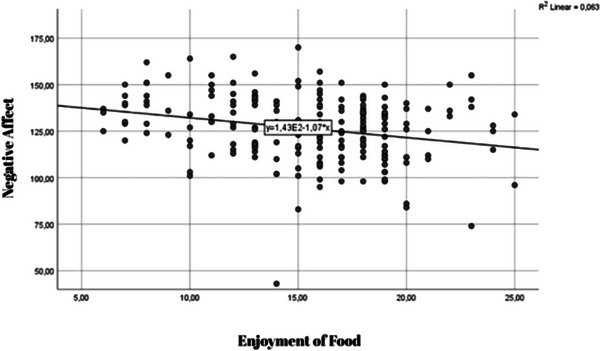
Scatter plot depicting the negative association between Enjoyment of Food and Negative Affect.

Figure 1, there is a noticeable trend suggesting that individuals who engage in high levels of EM also tend to exhibit increased patterns of EOE. The scatter plot presented in Figure  2 illustrates an inverse relationship between EF and negative emotional affect among the participants.

### 3.4 Multiple Regression Between Children's Eating Behavior and Temperament and Parent Feeding Behaviors

The multiple regression analysis predicting FR revealed that higher IF (*B* = 0.22, *p* = 0.028) and TIC (*B* = 0.27, *p* = 0.008) were significantly associated with greater FR scores. In contrast, TOC (*B* = −0.19, *p* = 0.046) was negatively associated with FR. Higher surgency (*B* = 0.03, *p* = 0.044) also predicted increased FR. Other predictors, including EM, encouragement feeding (EN), negative affect, effortful control, age, and sex, were not significant at the 0.05 level, though EN, negative affect, and age approached significance. The model explained 13% of the variance in FR (*R* ^2^ = 0.13) (Table 3).

**TABLE 3:** Spearman correlations between the subdimensions of the CEBQ and PFSQ.

Variables	EM	IF	EN	TİC	TOC
FR	0.155*	0.015	0.231**	0.083	−0.143**
EOE	0.316**	−0.158*	0.215**	−0.061	−0.168*
EF	−0.078	−0.004	−0.026	0.095	−0.007
DD	0.266**	0.003	0.216**	−0.045	−0.217**
SR	0.107	0.055	0.121	−0.062	−0.152*
SE	0.210**	0.139*	0.133	−0.020	−0.184**
EUE	0.227**	0.085	0.189**	0.054	−0.109
FF	0.003	0.117	0.009	0.076	−0.038

Abbreviations: DD: desire to drink, EF: enjoyment of food, EM: emotional feeding, EN: encouragement feeding, EOE: emotional overeating, EUE: emotional undereating, FF: food fussiness, FR: food responsiveness, IF: instrumental feeding, SE: slowness in eating, SR: satiety responsiveness, TIC: tightly controlled feeding, TOC: tolerance‐controlled feeding.**p < 0.01, *p < 0.05

The multiple regression analysis predicting EOE revealed that higher EM (*B* = 0.16, *p* = 0.003) was significantly associated with greater EOE scores. In contrast, EN (*B* = −0.12, *p* = 0.002) was negatively associated with EOE. Other predictors were not significant at the 0.05 level. The model explained 17% of the variance in EOE (*R* ^2^ = 0.17) (Table 4).

**TABLE 4:** Spearman's correlations between Child Eating Behavior Questionnaire (CEBQ) and Child Behavior Questionnaire (CBQ) subscales.

	CBQ		
CEBQ	Surgency	Negative affect	Effortful control
FR	0.061	−0.113	−0.087
EOE	−0.031	−0.019	−0.207**
EF	0.023	−0.255**	0.035
DD	0.177*	0.044	−0.141*
SR	0.226**	0.347**	−0.034
SE	−0.015	0.282**	−0.023
EUE	0.104	0.111	0.018
FF	−0.023	−0.225**	−0.062

Abbreviations: DD: desire to drink, EF: enjoyment of food, EOE: emotional overeating, EUE: emotional undereating, FF: food fussiness, FR: food responsiveness, SE: slowness in eating, SR: satiety responsiveness.

**p < 0.01, *p < 0.05

The multiple regression analysis predicting EF revealed that higher age (age; *B* = 0.12, p = 0.019) was significantly associated with greater EF scores. In contrast negative affect (Negative Affect; *B* = −0.09, *p* = 0.001) was negatively associated with EF. Other predictors were not significant at the 0.05 level. The model explained 13% of the variance in EF (*R* ^2^ = 0.13) (Table 5).

**TABLE 5:** Spearman's correlations between Child Eating Behavior Questionnaire (CEBQ) and Negative Affect subscales

CEBQ	Negative Affect				
	Discomfort	Fear	Anger/frustration	Sadness	Falling reactivity and soothability
FR	−0.129	−0.019	0.018	0.060	−0.136
EOE	−0.101	0.058	0.041	0.028	−0.016
EF	−0.232**	−0.133	−0.192**	−0.052	0.013
DD	−0.017	0.063	0.206**	0.065	−0.280**
SR	0.266**	0.169*	0.301**	0.291**	−0.180*
SE	0.241**	0.233**	0.116	0.127	−0.080
EUE	0.031	0.117	0.185**	0.093	−0.164*
FF	−0.180*	−0.121	−0.088	−0.158*	−0.058

**p < 0.01, *p < 0.05

The multiple regression analysis predicting DD revealed that higher surgency (surgency; *B* = 0.04, *p* = 0.001) was significantly associated with greater DD scores. In contrast TOC (*B* = −0.18, *p* = 0.032) was negatively associated with DD. Other predictors were not significant at the 0.05 level. The model explained 14% of the variance in DD (*R* ^2^ = 0.14) (Table 6).

**TABLE 6:** Spearman's correlations between Child Eating Behavior Questionnaire (CEBQ) and Surgency subscales

CEBQ	Surgency					
	Impulsivity	Activity level	Approach	High intensity pleasure	Smiling and laughter	Shyness
FR	0.067	0.002	−0.009	0.179*	0‐,078	0.042
EOE	0.112	0.001	−0.102	0.181**	−0.201**	−0.057
EF	0.022	0.075	−0.059	0.093	−0.004	−0.094
DD	0.089	0.228**	0.080	0.224**	−0.111	0.047
SR	0.136	0.209**	0.135	0.108	0.067	0.074
SE	0.015	−0.041	−0.003	0.001	0‐,025	0.006
EUE	0.041	0.048	0.123	0.108	−0.046	0.133
FF	0.002	0.001	−0.105	0.120	−0.085	−0.118

**p < 0.01, *p < 0.05

The multiple regression analysis predicting SR revealed that higher negative affect (Negative Affect; *B* = 0.10, *p* = 0.001) was significantly associated with greater SR scores. In contrast age (age; *B* = −1.14, *p* = 0.001) and effortful control (Effortful Control; *B* = −0.05, *p* = 0.009) were negatively associated with SR. Other predictors were not significant at the 0.05 level. The model explained 22% of the variance in SR (*R* ^2^ = 0.22) (**Table** **7**).

**TABLE 7:** Spearman's correlations between Child Eating Behavior Questionnaire (CEBQ) and Effortful Control subscales

CEBQ	Effortful Control			
	Low intensity pleasure	Inhibitory control	Perceptual sensitivity	Attentional focusing
FR	−0.067	−0.044	−0.047	−0.108
EOE	−0.211**	−0.101	−0.145*	−0.159*
EF	0.021	0.054	−0.049	0.068
DD	0.031	−0.210**	0.001	−0.186**
SR	0.127	−0.078	0.124	−0.229**
SE	0.034	−0.065	0.017	−0.041
EUE	0.123	−0.102	0.102	−0.059
FF	−0.055	−0.138*	−0.122	0.073

**p < 0.01, *p < 0.05

The multiple regression analysis predicting SE revealed that higher negative affect (Negative Affect; *B* = 0.08, *p* = 0.001) was significantly associated with greater SE scores. In contrast effortful control (Effortful Control; *B* = −0.01, *p* = 0.041) was negatively associated with SE. Other predictors were not significant at the 0.05 level. The model explained 19% of the variance in SE (*R* ^2^ = .19).

The multiple regression analysis predicting EUE revealed that higher EM (*B* = 0.17, *p* = 0.027) was significantly associated with greater EOE scores. Other predictors were not significant at the 0.05 level. The model explained 9% of the variance in EUE (*R* ^2^ = .09).

The multiple regression analysis predicting FF revealed that negative affect (Negative Affect; *B* = −0.04, *p* = 0.005) was negatively associated with FF. Other predictors were not significant at the 0.05 level. The model explained 9% of the variance in FF (*R* ^2^ = 0.09).

## 4 Discussion

This study aims to explore the relationship between children's eating behaviors, parental feeding behaviors, and child temperament among Turkish preschoolers aged 3–6 years.

### 4.1 Differences in Children's Eating Behaviors According to Sociodemographic Characteristics of Children and Families

Albuquerque et al. showed that children who had more siblings at the age of 4 years were less likely to develop SR, EF, SE and FF, which are considered as appetite restriction at the age of 7 years (Albuquerque et al. 2017). Having siblings has been shown to be a protective factor against the development of picky eating (Hafstad et al. 2013). In this study, no direct significant relationship was found between the number of siblings and eating behavior. Further studies in this field are needed.

In the study by Albuquerque et al., older maternal age and higher maternal education were found to be associated with higher EF, EOE, EUE, and DD (Albuquerque et al. 2017). In this study, higher maternal education level was associated with lower DD and SR in children. No significant relationship was found between maternal age and children's eating behavior.

In this study, no difference was found between children's eating behaviors according to the father's employment status, but significant differences were found according to the mother's employment status. It was shown that children of working mothers showed more food enjoyment and EE behaviors and less SR.

Another finding in our study was that there was no difference between the groups in terms of eating behavior characteristics according to income levels. This is similar to the results of Boswell et al. who found no difference in eating behavior in children compared according to income status (Boswell et al. 2018).

### 4.2 The Relationship Between Parental Feeding Style and Children's Eating Behaviors

One of the main focuses of this study is to examine the association between parental feeding styles and children's eating behaviors.

In a study conducted by Carnell et al. (2014), it was found that both parental IF and EM were associated with increased FR in children (Carnell et al. 2014). Similarly, in our study, consistent with previous research, we identified a relationship between parental feeding style and children's eating behaviors. Specifically, we found that parental EM was significantly associated with higher levels of EUE.

In the study conducted by Rodgers et al. (2013), EE (both EOE and EUE) showed a positive correlation with IF and EM practices, and a negative correlation with controlling feeding practices (Rodgers et al. 2013). Consistent with the findings of Rodgers et al. (2013), our study revealed similar results. We found a positive correlation between EUE in children EM practices in parents.

The “instrumental feeding” (i.e., using food as a reward) and “emotional feeding” (i.e., feeding in response to children's emotional distress) dimensions of parental feeding were positively associated with children's snacking behavior (Sleddens et al. 2010). In parallel with this study, IF and TIC were found to increase FR in our study. When food is used as a reward or incentive, children may begin to see food as a reward or a means of comfort. This can make food naturally more appealing and increase snacking behavior. These approaches may lead children to perceive food not only as nutrition but also as a means of reward, comfort, or control. In this case, children may find food more attractive and desire a variety of foods more. Furthermore, TIC can create resistance to restrictions in children and trigger overeating or snacking behaviors.

Analysis of the links between caregivers' feeding and children's eating behaviors revealed a finding that may be important. In our study, TOC served as a negative predictor for both DD and FR, indicating that higher levels of TOC are related to lower tendencies for these eating behaviors. These findings suggest that using food as a reward or as a means to alleviate negative emotions in children may have detrimental long‐term effects, including weight gain and the development of dysfunctional eating habits. Our results regarding EM and IF align with theories of eating disorders, which propose that excessive parental control over a child's food intake can disrupt hunger and satiety cues, leading to overeating in response to social or emotional situations (Birch et al. 2003). The findings from this study are significant as they support the notion that parental feeding practices may contribute to the development of various eating behaviors in children.

### 4.3 The Relationship Between Children's Temperament Traits and Eating Behaviors

In this study, we examined the relationship between three temperamental dimensions—surgency, negative affect, and effortful control—and eating behavior in preschool children. Our findings are partially consistent with previous research, providing further support for the association between child temperament characteristics and eating behaviors during the preschool period (Haycraft et al. 2011; Bjørklund et al. 2019).

Bjørklund et al. (2019) conducted a study demonstrating that the subdimension of negative affectivity characterized by lower levels of falling reactivity and higher levels of soothability predicted an increase in EOE over time (Bjørklund et al. 2019). Similarly, in our study, we found that negative affect temperament was negatively associated with EF and FF, indicating that higher levels of negative affect predict lower enjoyment and fussiness related to food. Conversely, negative affect was positively associated with SR and SE. We also found a negative association between EUE and lower levels of soothability, and a positive association with the anger/frustration temperament trait. Notably, SR exhibited a significant relationship with all negative affect temperament dimensions. Overall, it can be inferred that children with a difficult temperament (characterized by higher levels of negative affect) are less likely to employ constructive regulation strategies (Blair et al. 2004). These findings provide insights into the relationship between negative affect temperament and eating behaviors. Additionally, our study revealed an association between eating behavior, specifically SR, and the surgency temperament. We found that SR was positively correlated with the activity level within the surgency temperament subdimension, while it was negatively correlated with attentional focusing within the effortful control temperament subdimension.

The negative relationship we observed between the falling reactivity and soothability temperament subdimension and EUE in our study aligns with the findings of Haycraft et al. (2011). In their study, Haycraft et al. (2011) did not find associations between temperament traits such as shyness, sociability, and activity with any eating behaviors. However, they did find that high emotional temperament was linked to reduced EF and increased EE, SR, SE, and FF (Haycraft et al. 2011). Additionally, in the study conducted by Button et al. (2021), they demonstrated a positive relationship between children's FR and negative affect temperament, as well as a negative relationship between children's SE and the surgency temperament, which is significantly associated with impulsivity (Button et al. 2021). In contrast to the study conducted by Button et al. (2021), our findings indicate that FR was significantly associated solely with the high intensity pleasure temperament subdimension. Moreover, our study revealed that high intensity pleasure predicted greater FR. Additionally, we found that SE was significantly related to temperament characteristics of discomfort and fear, which are subdimensions of negative affect.

In the study conducted by Leung et al. (2014), it was found that higher levels of surgency were significantly associated with increased FR and EF (Leung et al. 2014). In our study, surgency was positively predictive of DD and FR, aligning with Leung et al.’s findings regarding its association with food‐related behaviors. However, an interesting divergence was observed in the relationship between surgency and eating slowness; in our results, higher surgency predicted slower eating, which contrasts with the typical expectation that higher surgency correlates with more impulsive or faster eating behaviors. This suggests that while surgency may increase responsiveness to food stimuli, its influence on eating pace might be more complex and context‐dependent. Further research could explore these differing patterns to better understand the multifaceted role of surgency in eating behaviors.

These factors highlight the complexity of studying the relationship between personality traits and eating behaviors. Understanding these nuances can enhance the interpretation of our findings and their implications in the field.

Surgency, which is significantly associated with impulsivity and hyperactivity, may influence the development of disordered eating behaviors that contribute to childhood nutritional disorders. The disparities between the findings of these studies may be attributed to differences in sample selection. Leung et al. (2014) focused on children from low socioeconomic backgrounds, whereas our study included a sample from the general population. It has been reported that preschool children from low‐income families tend to consume more food, eat more frequently, derive greater pleasure from eating, and engage in eating behaviors in the absence of hunger in response to external food cues (Leung et al. 2014).

These insights are essential, as the early years of a child's life are formative. Establishing healthy eating patterns during preschool can have long‐lasting effects on children's dietary choices in later years, potentially reducing the risk of obesity and related health issues. By understanding these dynamics, parents and caregivers can make informed decisions that promote not only immediate health benefits but also contribute to lifelong positive eating habits (Dulude 2011; Maffeis 2014; Ruel and Hoddinott 2008).

Healthcare professionals and therapists working with children and families can use the information regarding the relationship between parental feeding styles and children's eating behaviors to develop personalized intervention strategies. For instance, if EM practices are identified as problematic, clinicians can work with parents to develop alternative ways of addressing their children's emotional needs without using food. Likewise, if TIC practices are negatively associated with children's eating behaviors, alternative, less controlling approaches can be recommended. Recognizing the connection between children's temperamental traits and eating behaviors can help healthcare providers identify children at risk of developing problematic eating behaviors early on. This information can guide early intervention and support for these children and their families, potentially preventing long‐term issues related to eating disorders and unhealthy eating habits. This knowledge can be used to implement preventive measures to reduce the risk of eating disorders and obesity in children.

## 5 Limitations

The study has several limitations. The sample size is relatively small, which may limit the generalizability of the findings. The research design is not observational, which could affect the accuracy of the measurements. The data collected relies on parents' self‐reports, which may introduce bias or inaccuracies and the absence of objective height and weight measurements. The participants were selected only from the central districts of Ankara city, which may restrict the representativeness of the sample. There is not much diversity in education or family structure. The cross‐sectional nature of the study prevents determining whether the “predictors” (temperament and parenting) lead to the outcome (child eating behavior) or vice versa. In other words, this type of study collects data at a specific point in time and does not allow for establishing cause‐and‐effect relationships definitively. The fact that parents reported on all three constructs of interest within the same questionnaire: This approach can introduce common‐method variance or spurious correlations due to measurement issues. In other words, having the same person report on all three constructs may lead to relationships that are more a result of the reporting method itself rather than true associations.

Limitations of the study also include the lack of examination of cultural influences on parental eating habits and the omission of gender differences in outcomes, despite the potential importance of differences in eating behaviors and temperament between boys and girls

## 6 Conclusions

Our findings revealed that parental feeding behaviors and temperamental traits significantly influence children's eating behaviors. Understanding the connections between eating behaviors, temperamental traits, and parental feeding styles at an early age can be valuable for clinicians and healthcare professionals working with children facing feeding difficulties. In particular, suboptimal parental feeding styles and challenging temperaments in children have a significant impact on eating behavior. The results obtained from this study hold significant implications in this regard.

## Author Contributions

**İbrahim Zeyrek**: conceptualization, methodology, writing–original draft, writing–review and editing, investigation, validation, visualization, software, data curation, resources. **Esra Güney**: conceptualization, methodology, writing–review and editing, writing–original draft, supervision, project administration. **Ahmet Özaslan**: conceptualization, writing–original draft, writing–review and editing, formal analysis, supervision, methodology. **Asiye Uğraş Dikmen**: writing–original draft, writing–review and editing, formal analysis, methodology, supervision.

## Ethics Statement

This study received approval from the Research Ethics Committee Of Gazi University. Prior to the collection of any information, online informed consent was obtained from all participating parents.

## Consent

All parents were fully aware of and consented to the publication of their information in any journal.

## Conflicts of Interest

The authors declare no conflicts of interest.

## Peer Review

The peer review history for this article is available at https://publons.com/publon/10.1002/brb3.70758

## Supporting information




**Supporting Material**: brb370758‐sup‐0001‐SuppMat.docx

## Data Availability

The data that support the findings of this study are available from the corresponding author upon reasonable request.

## References

[brb370758-bib-0001] Agras, W. S. , L. D. Hammer , F. McNicholas , and H. C. Kraemer . 2004. “Risk Factors for Childhood Overweight: A Prospective Study From Birth to 9.5 Years.” Journal of Pediatrics 145, no. 1: 20–25. 15238901 10.1016/j.jpeds.2004.03.023

[brb370758-bib-0002] Albuquerque, G. , M. Severo , and A. Oliveira . 2017. “Early Life Characteristics Associated With Appetite‐Related Eating Behaviors in 7‐Year‐Old Children.” Journal of Pediatrics 180: 38–46.e2. 27769552 10.1016/j.jpeds.2016.09.011

[brb370758-bib-0003] Anzman‐Frasca, S. , C. A. Stifter , and L. L. Birch . 2012. “Temperament and Childhood Obesity Risk: A Review of the Literature.” Journal of Developmental & Behavioral Pediatrics 33, no. 9: 732–745. 23095495 10.1097/DBP.0b013e31826a119f

[brb370758-bib-0004] Benjasuwantep, B. , S. Chaithirayanon , and M. Eiamudomkan . 2013. “Feeding Problems in Healthy Young Children: Prevalence, Related Factors and Feeding Practices.” Pediatric Reports 5, no. 2: e10. 10.4081/pr.2013.e10 PMC3718228 23904965

[brb370758-bib-0005] Bergmeier, H. , H. Skouteris , and M. Hetherington . 2015. “Systematic Research Review of Observational Approaches Used to Evaluate Mother‐Child Mealtime Interactions During Preschool Years.” American Journal of Clinical Nutrition 101, no. 1: 7–15. 25527745 10.3945/ajcn.114.092114

[brb370758-bib-0006] Bergmeier, H. , H. Skouteris , S. Horwood , M. Hooley , and B. Richardson . 2014. “Associations Between Child Temperament, Maternal Feeding Practices and Child Body Mass Index During the Preschool Years: A Systematic Review of the Literature.” Obesity Reviews 15, no. 1: 9–18. 23957249 10.1111/obr.12066

[brb370758-bib-0007] Birch, L. L. , J. O. Fisher , and K. K. Davison . 2003. “Learning to Overeat: Maternal Use of Restrictive Feeding Practices Promotes Girls' Eating in the Absence of Hunger.” American Journal of Clinical Nutrition 78, no. 2: 215–220. 12885700 10.1093/ajcn/78.2.215 PMC2530927

[brb370758-bib-0008] Birch, L. L. , J. O. Fisher , K. Grimm‐Thomas , C. N. Markey , R. Sawyer , and S. L. Johnson . 2001. “Confirmatory Factor Analysis of the Child Feeding Questionnaire: A Measure of Parental Attitudes, Beliefs and Practices About Child Feeding and Obesity Proneness.” Appetite 36, no. 3: 201–210. 11358344 10.1006/appe.2001.0398

[brb370758-bib-0009] Bjørklund, O. , L. Wichstrøm , C. H. Llewellyn , and S. Steinsbekk . 2019. “Emotional Over‐and Undereating in Children: A Longitudinal Analysis of Child and Contextual Predictors.” Child Development 90, no. 6: e803–e818. 29959767 10.1111/cdev.13110

[brb370758-bib-0010] Blair, K. A. , S. A. Denham , A. Kochanoff , and B. Whipple . 2004. “Playing It Cool: Temperament, Emotion Regulation, and Social Behavior in Preschoolers.” Journal of School Psychology 42, no. 6: 419–443.

[brb370758-bib-0011] Blissett, J. , and C. Farrow . 2007. “Predictors of Maternal Control of Feeding at 1 and 2 Years of Age.” International Journal of Obesity 31, no. 10: 1520–1526. 17579636 10.1038/sj.ijo.0803661

[brb370758-bib-0012] Boswell, N. , R. Byrne , and P. S. Davies . 2018. “Eating Behavior Traits Associated With Demographic Variables and Implications for Obesity Outcomes in Early Childhood.” Appetite 120: 482–490. 29024677 10.1016/j.appet.2017.10.012

[brb370758-bib-0013] Braet, C. , L. Claus , S. Verbeken , and L. Van Vlierberghe . 2007. “Impulsivity in Overweight Children.” European Child & Adolescent Psychiatry 16: 473–483. 17876511 10.1007/s00787-007-0623-2

[brb370758-bib-0014] Button, A. , M. S. Faith , and R. I. Berkowitz . 2021. “Temperament and Eating Self‐Regulation in Young Children With or at Risk for Obesity: An Exploratory Report.” Pediatric Obesity 16, no. 11: e12821. 34080805 10.1111/ijpo.12821

[brb370758-bib-0015] Carnell, S. , L. Benson , E. Driggin , and L. Kolbe . 2014. “Parent Feeding Behavior and Child Appetite: Associations Depend on Feeding Style.” International Journal of Eating Disorders 47, no. 7: 705–709. 24976396 10.1002/eat.22324 PMC4211951

[brb370758-bib-0016] de Sousa Maranhão, H. , R. C. de Aguiar , D. T. J. de Lira , M. Ú. F. Sales , and N. Á. do Nascimento Nóbrega . 2017. “Feeding Difficulties in Preschool Children, Previous Feeding Practices, and Nutritional Status.” Revista Paulista De Pediatria 36: 45–51. 10.1590/1984-0462/;2018;36;1;00004 PMC5849369 29091129

[brb370758-bib-0017] Dulude, G. 2011. "Relations entre le style parental, le style parental alimentaire et les pratiques alimentaires de la mère et les comportements alimentaires de l'enfant québécois d’âge préscolaire." PhD diss., Université de Montréal.

[brb370758-bib-0018] Farrow, C. , and J. Blissett . 2006. “Maternal Cognitions, Psychopathologic Symptoms, and Infant Temperament as Predictors of Early Infant Feeding Problems: A Longitudinal Study.” International Journal of Eating Disorders 39, no. 2: 128–134. 16231348 10.1002/eat.20220

[brb370758-bib-0019] Green, S. B. 1991. “How Many Subjects Does It Take to Do a Regression Analysis.” Multivariate Behavioral Research 26, no. 3: 499–510. 26776715 10.1207/s15327906mbr2603_7

[brb370758-bib-0020] Gregory, J. E. , S. J. Paxton , and A. M. Brozovic . 2010. “Maternal Feeding Practices, Child Eating Behaviour and Body Mass Index in Preschool‐Aged Children: A Prospective Analysis.” International Journal of Behavioral Nutrition and Physical Activity 7, no. 1: 1–10. 20579397 10.1186/1479-5868-7-55 PMC2907299

[brb370758-bib-0021] Hafstad, G. S. , D. S. Abebe , L. Torgersen , and T. von Soest . 2013. “Picky Eating in Preschool Children: The Predictive Role of the Child's Temperament and Mother's Negative Affectivity.” Eating Behaviors 14, no. 3: 274–277. 23910765 10.1016/j.eatbeh.2013.04.001

[brb370758-bib-0022] Haycraft, E. , C. Farrow , C. Meyer , F. Powell , and J. Blissett . 2011. “Relationships Between Temperament and Eating Behaviours in Young Children.” Appetite 56, no. 3: 689–692. 21316412 10.1016/j.appet.2011.02.005

[brb370758-bib-0023] Juanjuan, L. 2017. “Influencing Factors and Prevention Strategies of Obesity in Preschool Children.” Shanghai Medical & Pharmaceutical Journal 38, no. 02: 59–62.

[brb370758-bib-0024] Larsen, J. K. , R. C. Hermans , E. F. Sleddens , R. C. Engels , J. O. Fisher , and S. P. Kremers . 2015. “How Parental Dietary Behavior and Food Parenting Practices Affect Children's Dietary Behavior. Interacting Sources of Influence?” Appetite 89: 246–257. 25681294 10.1016/j.appet.2015.02.012

[brb370758-bib-0025] Leung, C. Y. , J. C. Lumeng , N. A. Kaciroti , Y. P. Chen , K. Rosenblum , and A. L. Miller . 2014. “Surgency and Negative Affectivity, but Not Effortful Control, Are Uniquely Associated With Obesogenic Eating Behaviors Among Low‐Income Preschoolers.” Appetite 78: 139–146. 24685763 10.1016/j.appet.2014.03.025 PMC4039349

[brb370758-bib-0026] Lobstein, T. , L. Baur , and R. Uauy . 2004. “Obesity in Children and Young People: A Crisis in Public Health.” Obesity Reviews 5: 4–85. 15096099 10.1111/j.1467-789X.2004.00133.x

[brb370758-bib-0027] Maffeis, C. 2014. “Early Prevention of Obesity.” Journal of Pediatric and Neonatal Individualized Medicine (JPNIM) 3, no. 2: e030250‐e.

[brb370758-bib-0028] McMeekin, S. , E. Jansen , K. Mallan , J. Nicholson , A. Magarey , and L. Daniels . 2013. “Associations Between Infant Temperament and Early Feeding Practices. A Cross‐Sectional Study of Australian Mother‐Infant Dyads From the NOURISH Randomised Controlled Trial.” Appetite 60: 239–245. 23079142 10.1016/j.appet.2012.10.005

[brb370758-bib-0029] Messerli‐Bürgy, N. , K. Stülb , T. H. Kakebeeke , et al. 2018. “Emotional Eating Is Related With Temperament but Not With Stress Biomarkers in Preschool Children.” Appetite 120: 256–264. 28866031 10.1016/j.appet.2017.08.032

[brb370758-bib-0030] Morin, K. H. 2006. “Parental Style of Infant and Child Feeding: How Influential Is It?.” MCN: The American Journal of Maternal/Child Nursing 31, no. 6: 388. 17149116 10.1097/00005721-200611000-00011

[brb370758-bib-0031] Nowicka, P. , K. Sorjonen , A. Pietrobelli , C.‐E. Flodmark , and M. S. Faith . 2014. “Parental Feeding Practices and Associations With Child Weight Status. Swedish Validation of the Child Feeding Questionnaire Finds Parents of 4‐Year‐Olds Less Restrictive.” Appetite 81: 232–241. 24972134 10.1016/j.appet.2014.06.027

[brb370758-bib-0032] Özçetin, M. , R. Yılmaz , Ü. Erkorkmaz , and H. Esmeray . 2010. “Ebeveyn besleme Tarzı Anketi Geçerlik Ve Güvenirlik Çalışması.” Turkish Archives of Pediatrics 45, no. 2: 24–31.

[brb370758-bib-0033] Pliner, P. , and E. R. Loewen . 1997. “Temperament and Food Neophobia in Children and Their Mothers.” Appetite 28, no. 3: 239–254. 9218097 10.1006/appe.1996.0078

[brb370758-bib-0034] Putnam, S. P. , and M. K. Rothbart . 2006. “Development of Short and Very Short Forms of the Children's Behavior Questionnaire.” Journal of Personality Assessment 87, no. 1: 102–112. 16856791 10.1207/s15327752jpa8701_09

[brb370758-bib-0035] Rodgers, R. F. , S. J. Paxton , R. Massey , et al. 2013. “Maternal Feeding Practices Predict Weight Gain and Obesogenic Eating Behaviors in Young Children: A Prospective Study.” International Journal of Behavioral Nutrition and Physical Activity 10, no. 1: 1–10. 23414332 10.1186/1479-5868-10-24 PMC3582584

[brb370758-bib-0036] Rothbart, M. K. , S. A. Ahadi , and D. E. Evans . 2000. “Temperament and Personality: Origins and Outcomes.” Journal of Personality and Social Psychology 78, no. 1: 122–135. 10653510 10.1037//0022-3514.78.1.122

[brb370758-bib-0037] Rothbart, M. K. , and S. P. Putnam . 2002. “Temperament and Socialization.” In Paths to Successful Development: Personality in the Life Course, edited by L. Pulkkinen and A. Caspi , 19–45. Cambridge University Press.

[brb370758-bib-0038] Ruel, M. T. , and J. F. Hoddinott . 2008. Investing in Early Childhood Nutrition. IFPRI.

[brb370758-bib-0039] Sarı, B. A. , E. İşeri , Ö. Yalçın , A. A. Aslan , and Ş. Şener . 2012. “Çocuk Davranış Listesi Kısa Formunun Türkçe Güvenilirlik Çalışması Ve Geçerliliğine İlişkin Ön Çalışma.” Klinik Psikiyatri Dergisi 15, no. 3: 135–143.

[brb370758-bib-0040] Shields, A. , and D. Cicchetti . 1997. “Emotion Regulation Among School‐Age Children: The Development and Validation of a New Criterion Q‐Sort Scale.” Developmental Psychology 33, no. 6: 906–916. 9383613 10.1037//0012-1649.33.6.906

[brb370758-bib-0041] Shloim, N. , L. R. Edelson , N. Martin , and M. M. Hetherington . 2015. “Parenting Styles, Feeding Styles, Feeding Practices, and Weight Status in 4–12 Year‐Old Children: A Systematic Review of the Literature.” Frontiers in Psychology 6: 1849. 26696920 10.3389/fpsyg.2015.01849 PMC4677105

[brb370758-bib-0042] Sleddens, E. F. C. , S. O. Hughes , T. M. O'Connor , et al. 2012. “The Children's Behavior Questionnaire Very Short Scale: Psychometric Properties and Development of a One‐Item Temperament Scale.” Psychological Reports 110, no. 1: 197–217. 22489386 10.2466/08.10.21.PR0.110.1.197-217

[brb370758-bib-0043] Sleddens, E. F. C. , S. P. Kremers , N. K. De Vries , and C. Thijs . 2010. “Relationship Between Parental Feeding Styles and Eating Behaviours of Dutch Children Aged 6–7.” Appetite 54, no. 1: 30–36. 19747513 10.1016/j.appet.2009.09.002

[brb370758-bib-0044] Ünlü, H. 2011. Okul öncesi dönem çocuklar Için yeme davranışı Değerlendirme Ölçeğinin Türk Çocuklarına uyarlanması. Marmara Üniversitesi Eğitim Bilimleri Enstitüsü, Yüksek Lisans Tezi.

[brb370758-bib-0045] Vollrath, M. E. , S. Tonstad , M. K. Rothbart , and S. E. Hampson . 2011. “Infant Temperament Is Associated With Potentially Obesogenic Diet at 18 Months.” International Journal of Pediatric Obesity 6, no. sup3: e408–e414. 20854098 10.3109/17477166.2010.518240 PMC3128685

[brb370758-bib-0046] Wardle, J. , C. A. Guthrie , S. Sanderson , and L. Rapoport . 2001. “Development of the Children's Eating Behaviour Questionnaire.” Journal of Child Psychology and Psychiatry and Allied Disciplines 42, no. 7: 963–970. 11693591 10.1111/1469-7610.00792

[brb370758-bib-0047] Wardle, J. , S. Sanderson , C. A. Guthrie , L. Rapoport , and R. Plomin . 2002. “Parental Feeding Style and the Inter‐Generational Transmission of Obesity Risk.” Obesity Research 10, no. 6: 453–462. 12055321 10.1038/oby.2002.63

[brb370758-bib-0048] Webber, L. , L. Cooke , C. Hill , and J. Wardle . 2010. “Associations Between Children's Appetitive Traits and Maternal Feeding Practices.” Journal of the American Dietetic Association 110, no. 11: 1718–1722. 21034886 10.1016/j.jada.2010.08.007

[brb370758-bib-0049] Yılmaz, R. , H. Esmeray , and Ü. Erkorkmaz . 2011. “Çocuklarda Yeme Davranışı Anketinin Türkçe uyarlama çalışması.” Anatolian Journal of Psychiatry/Anadolu Psikiyatri Dergisi 12, no. 4: 287–294.

[brb370758-bib-0050] Yuan, J. , Y. Zhang , Z. Lu , et al. 2019. “Correlation Between Children's Eating Behaviors and Caregivers' Feeding Behaviors Among Preschool Children in China.” Appetite 138: 146–152. 30917942 10.1016/j.appet.2019.03.022

