# Supplementary material for: Eating Behavior in Children Aged 3–6: Relationship With the Child's Temperament Characteristics and Parent's Feeding Style
Source: Brain Behav. 2025 Aug 12;15(8):e70758. doi: 10.1002/brb3.70758 (PMC12340540; doi:10.1002/brb3.70758)
Supplement: Supplementary file 1 — Supporting Material: brb370758‐sup‐0001‐SuppMat.docx [file BRB3-15-e70758-s001.docx]

**SUPPLEMENTARY TABLES**

The results of the Spearman correlation test are shown in Table 1.

In the study, the correlations between 8 variables from the CEBQ and 5 variables from the PFSQ were examined. The analysis revealed a significant and moderate positive correlation between the desire to drink subscale score and the emotional feeding subscale score (r = 0.266, p < 0.01). Additionally, a moderate positive correlation was observed between the emotional overeating subscale and the emotional feeding subscale (r = 0.316, p < 0.01). However, low correlations were found within and between the remaining CEBQ and PFSQ variables.

| **Table 1**  Spearman Correlations Between the Subdimensions of the CEBQ and PFSQ. | | | | | |
| --- | --- | --- | --- | --- | --- |
|  | PFSQ | | | | |
| CEBQ | EM | IF | EN | TİC | TOC |
| FR | 0,155* | 0,015 | 0,231** | 0,083 | -0,143** |
| EOE | 0,316** | -0,158* | 0,215** | -0,061 | -0,168* |
| EF | -0,078 | -0,004 | -0,026 | 0,095 | -0,007 |
| DD | 0,266** | 0,003 | 0,216** | -0,045 | -0,217** |
| SR | 0,107 | 0,055 | 0,121 | -0,062 | -0,152* |
| SE | 0,210** | 0,139* | 0,133 | -0,020 | -0,184** |
| EUE | 0,227** | 0,085 | 0,189** | 0,054 | -0,109 |
| FF | 0,003 | 0,117 | 0,009 | 0,076 | -0,038 |
| **p<0,01, *p<0,05  FR: food responsiveness, EOE: emotional over eating, EF: enjoyment of food, DD: desire to drink, SR: satiety responsiveness, SE: slowness in eating, EUE: emotional undereating, FF: food fussiness  EM: emotional feeding, IF: instrumental feeding, EN: encouragement feeding, TİC: tightly-controlled feeding, TOC: tolerance controlled feeding | | | | | |

Spearman correlations between CBQ and CEBQ subscales, are provided in Table 2.

In our study, we observed several correlations between different variables. We found a positive and low correlation between surgency and the desire to drink subscale (r = 0.177, p = 0.012), as well as between surgency and the satiety responsiveness subscale (r = 0.226, p = 0.001). On the other hand, a negative and moderate relationship was identified between negative affect and the enjoyment of food subscale (r = -0.255, p < 0.001). Furthermore, we found a negative and low correlation between negative affect and the food fussiness subscale (r = -0.225, p = 0.001). Additionally, a positive and moderate correlation was observed between negative affect and the satiety responsiveness subscale (r = 0.347, p < 0.001), as well as between negative affect and the slowness in eating subscale (r = 0.282, p < 0.001). Lastly, we determined a negative and low correlation between effortful control and emotional overeating subscale (r = -0.207, p = 0.003), as well as between effortful control and the desire to drink subscale (r = -0.141, p = 0.044).

| **Table 2**  Spearman's Correlations Between Child Eating Behaviour Questionnaire (CEBQ) and Child Behaviour Questionnaire (CBQ) Subscales | | | |
| --- | --- | --- | --- |
|  | CBQ | | |
| CEBQ | Surgency | Negative affect | Effortful control |
| FR | 0,061 | -0,113 | -0,087 |
| EOE | -0,031 | -0,019 | -0,207** |
| EF | 0,023 | -0,255** | 0,035 |
| DD | 0,177* | 0,044 | -0,141* |
| SR | 0,226** | 0,347** | -0,034 |
| SE | -0,015 | 0,282** | -0,023 |
| EUE | 0,104 | 0,111 | 0,018 |
| FF | -0,023 | -0,225** | -0,062 |
| **p<0,01, *p<0,05  FR: Food responsiveness, EOE: emotional over eating, EF: enjoyment of food, DD: Desire to drink, SR: Satiety responsiveness, SE: Slowness in eating, EUE: Emotional undereating, FF: Food fussiness | | | |

The results of the Spearman correlation test between the Children's Eating Behaviour Questionnaire (CEBQ) and negative affect subscales are presented in Table 5. We observed a moderate and negative relationship between the desire to drink and falling reactivity and soothability (r = -0.280, p < 0.001). Additionally, we found a moderate and positive correlation between satiety responsiveness and discomfort (r = 0.266, p < 0.001), anger/frustration (r = 0.301, p < 0.001), and sadness (r = 0.291, p < 0.001). For more correlation results, please refer to Table 3.

| **Table 3**  Spearman's Correlations Between Child Eating Behaviour Questionnaire (CEBQ) and Negative Affect Subscales | | | | | |
| --- | --- | --- | --- | --- | --- |
| CEBQ | Negative Affect | | | | |
|  | Discomfort | Fear | Anger/frustration | Sadness | Falling Reactivity and Soothability |
| FR | -0,129 | -0,019 | 0,018 | 0,060 | -0,136 |
| EOE | -0,101 | 0,058 | 0,041 | 0,028 | -0,016 |
| EF | -0,232** | -0,133 | -0,192** | -0,052 | 0,013 |
| DD | -0,017 | 0,063 | 0,206** | 0,065 | -0,280** |
| SR | 0,266** | 0,169* | 0,301** | 0,291** | -0,180* |
| SE | 0,241** | 0,233** | 0,116 | 0,127 | -0,080 |
| EUE | 0,031 | 0,117 | 0,185** | 0,093 | -0,164* |
| FF | -0,180* | -0,121 | -0,088 | -0,158* | -0,058 |
| **p<0,01, *p<0,05 | | |  |  |  |

The results of the Spearman correlation test are displayed in Table 4. Within the CEBQ and surgency variables, low correlations were observed. Among the significant relationships, only a negative and low correlation was found between emotional overeating and the temperament trait of smiling and laughter (r = -0.201, p = 0.004). For all other significant variables, positive and low correlations were identified

| **Table 4**  Spearman's Correlations Between Child Eating Behaviour Questionnaire (CEBQ) and Surgency Subscales | | | | | | | |
| --- | --- | --- | --- | --- | --- | --- | --- |
| CEBQ | Surgency | | | | | | |
|  |  | Impulsivity | Activity Level | Approach | High Intensity Pleasure | Smiling and Laughter | Shyness |
| FR |  | 0,067 | 0,002 | -0,009 | 0,179* | 0-,078 | 0,042 |
| EOE |  | 0,112 | 0,001 | -0,102 | 0,181** | -0,201** | -0,057 |
| EF |  | 0,022 | 0,075 | -0,059 | 0,093 | -0,004 | -0,094 |
| DD |  | 0,089 | 0,228** | 0,080 | 0,224** | -0,111 | 0,047 |
| SR |  | 0,136 | 0,209** | 0,135 | 0,108 | 0,067 | 0,074 |
| SE |  | 0,015 | -0,041 | -0,003 | 0,001 | 0-,025 | 0,006 |
| EUE |  | 0,041 | 0,048 | 0,123 | 0,108 | -0,046 | 0,133 |
| FF |  | 0,002 | 0,001 | -0,105 | 0,120 | -0,085 | -0,118 |
| **p<0,01, *p<0,05 | | |  |  |  |  |  |

Correlations between the CEBQ variables and effortful control variables are shown in Table 5.

In examining the relationship between child eating behavior and the temperament of effortful control, we found a negative and low correlation between emotional overeating and the temperament characteristics of low intensity pleasure (r = -0.211, p = 0.003), perceptual sensitivity (r = -0.145, p = 0.038), and attentional focusing (r = -0.159, p = 0.023). Furthermore, other significant results also revealed low and negative correlations.

| **Table 5**  Spearman's Correlations Between Child Eating Behaviour Questionnaire (CEBQ) and Effortful Control Subscales | | | | |
| --- | --- | --- | --- | --- |
| CEBQ | Effortful Control | | | |
|  | Low Intensity Pleasure | Inhibitory Control | Perceptual Sensitivity | Attentional Focusing |
| FR | -0,067 | -0,044 | -0,047 | -0,108 |
| EOE | -0,211** | -0,101 | -0,145* | -0,159* |
| EF | 0,021 | 0,054 | -0,049 | 0,068 |
| DD | 0,031 | -0,210** | 0,001 | -0,186** |
| SR | 0,127 | -0,078 | 0,124 | -0,229** |
| SE | 0,034 | -0,065 | 0,017 | -0,041 |
| EUE | 0,123 | -0,102 | 0,102 | -0,059 |
| FF | -0,055 | -0,138* | -0,122 | 0,073 |
| **p<0,01, *p<0,05 | | |  |  |
|  | | |  |  |
